# Comprehensive Two-Dimensional Liquid Chromatography–High-Resolution Mass Spectrometry for Complex Protein Digest Analysis Using Parallel Gradients

**DOI:** 10.1021/acs.analchem.4c02172

**Published:** 2024-05-17

**Authors:** Rick S. van den Hurk, Bart Lagerwaard, Nathan J. Terlouw, Mingzhe Sun, Job J. Tieleman, Anniek X. Verstegen, Saer Samanipour, Bob W.J. Pirok, Andrea F.G. Gargano

**Affiliations:** † Analytical Chemistry Group, Van’t Hoff Institute for Molecular Sciences, University of Amsterdam, Amsterdam1098 XH,The Netherlands; ‡ Centre for Analytical Sciences Amsterdam (CASA), Amsterdam1098 XH,The Netherlands

## Abstract

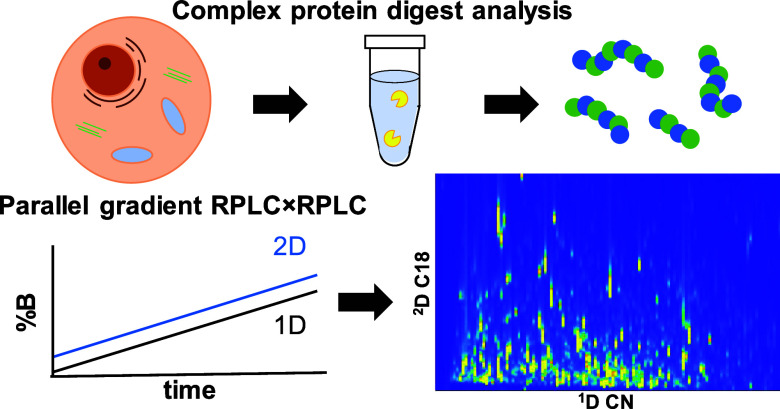

Despite the high gain in peak capacity, online comprehensive two-dimensional liquid chromatography coupled with high-resolution mass spectrometry (LC × LC-HRMS) has not yet been widely applied to the analysis of complex protein digests. One reason is the method's reduced sensitivity which can be linked to the high flow rates of the second separation dimension (^2^D). This results in higher dilution factors and the need for flow splitters to couple to ESI-MS. This study reports proof-of-principle results of the development of an RPLC × RPLC-HRMS method using parallel gradients (^2^D flow rate of 0.7 mL min^–1^) and its comparison to shifted gradient methods (^2^D of 1.4 mL min^–1^) for the analysis of complex digests using HRMS (QExactive-Plus MS). Shifted and parallel gradients resulted in high surface coverage (SC) and effective peak capacity (SC of 0.6226 and 0.7439 and effective peak capacity of 779 and 757 in 60 min). When applied to a cell line digest sample, parallel gradients allowed higher sensitivity (e.g., average MS intensity increased by a factor of 3), allowing for a higher number of identifications (e.g., about 2600 vs 3900 peptides). In addition, reducing the modulation time to 10 s significantly increased the number of MS/MS events that could be performed. When compared to a 1D-RPLC method, parallel RPLC × RPLC-HRMS methods offered a higher separation performance (FHWH from 0.12 to 0.018 min) with limited sensitivity losses resulting in an increase of analyte identifications (e.g., about 6000 vs 7000 peptides and 1500 vs 1990 proteins).

## 1 Introduction

Modern liquid chromatography (LC)-high-resolution mass spectrometry (HRMS) instruments reach scan rates of over 50 Hz, allowing for fast analysis and fragmentation experiments of peptides sequences. This makes LC-HRMS the method of choice to study changes in the proteome of complex organisms and to characterize the sequence of proteins, such as biotherapeutics.^1−3^ In these experiments, proteins are digested into peptides, and LC separations are essential to resolve the tens of thousands of peptides in a sample. The separation quality thus significantly influences the speed and depth of this analysis.^5^ The metric most often used to describe the quality of an LC separation is the peak capacity, approximating the maximum number of peaks that can be resolved at an equal resolution within a given separation space.^6^ Ultrahigh-pressure LC technology commonly allows for peak capacity between 100 and 200 per hour.^7^ For the analysis of highly complex samples, comprehensive two-dimensional LC (LC × LC) is an attractive option as it can offer 1 order of magnitude higher peak capacity.^8−11^

In the past years, LC × LC has been applied for separating protein digests and other peptide mixtures. A commonly applied selectivity combination is RPLC × RPLC.^12^ This combination yields fundamentally limited orthogonality yet provides excellent solvent compatibility between the dimensions and high-resolution separations. The most common methods either employ different column chemistries (e.g., ref (13)) or combine basic mobile phases in the first dimension (^1^D) with acidic RPLC in the second dimension (^2^D; e.g., ref (4)). Nevertheless, the limited orthogonality of the two methods results in low retention-space utilization when using full gradients (the same gradient in every ^2^D separation, e.g., 2–45%B). For this reason, shifted gradients (the lower and upper boundary of the ^2^D gradient change) can be used where the ^2^D mobile phase gradient method is correlated to the gradient program in ^1^D to maximize the surface coverage.^9^ Using this approach, Stoll et al. reached a peak capacity of 10,000 in 4 h for the analysis of a monoclonal antibody digest.^4^ Despite high performance, the use of shifted gradients has also been criticized. Chapel et al.^14^ found that the increase in retention space coverage and peak capacity is obtained at the expense of sensitivity and retention time repeatability in consecutive ^2^D separations.

Moreover, the most critical disadvantage of any repeating gradient (i.e., shifted or full) in RPLC × RPLC is that high flow rates (>1 mL min^–1^) are required to minimize dwell time, column equilibration time, and *t*_0_ along with increasing the normalized gradient slope and overall separation power. However, high flow rates increase the dilution factors and require the use of postcolumn flow splitting to allow for hyphenation with MS, inducing band distortion and losses in sensitivity.^15^

One alternative to shifted gradients to extend the usage of the ^2^D retention space in RPLC × RPLC is using parallel gradients. With this approach, in the second-dimension separation, a single gradient with a slope correlated to the first dimension (hence “parallel”) is programmed throughout the analysis. Parallel gradients have been investigated since 2003,^16^ demonstrating that this method can improve the use of available ^2^D separation space in correlated RPLC × RPLC platforms.^17−20^ An additional advantage is more constant pressure on the ^2^D column, reducing physical stress on the column and other system components.^21^ Moreover, parallel gradients do not require high ^2^D flow rates and consequently omit the need for postcolumn flow splitting when hyphenating to MS. Various applications have been demonstrated with these methods, such as pharmaceuticals analysis,^20^ food,^19,22^ and simple aromatic compounds.^19^

In this work, we developed a parallel-gradient RPLC × RPLC method for peptide separations and compared it to full- and shifted gradients. All methods employed stationary-phase-assisted modulation (SPAM) as a modulation strategy.^23−255^ Our comparison was based on the effective peak capacity, sensitivity, surface coverage, and repeatability. Finally, the methods were evaluated based on their protein-identification capacity by measuring a cell lysate using data depended MS/MS. Short modulation times (30, 20 and 10 s) for parallel-gradient methods were also explored.

## 2 Experimental Section

### 2.1 Chemicals

Water (ULC/MS grade) and acetonitrile (ACN, LC-MS grade) were obtained from Biosolve (Valkenswaard, The Netherlands). Dichloromethane (DCM) was obtained from VWR chemicals (Fontenay-sous-Bois, France). Ammonium bicarbonate (Bioultra, ≥99.5%) was acquired from Fluka Analytical (Charlotte, USA). Formic acid (FA, ≥98%) and ammonium formate (AmFm) were obtained from Sigma (Zwijndrecht, The Netherlands). The ammonium hydroxide solution was obtained from Thermoscientific (The Netherlands).

Alpha casein (≥70.0%), bovine serum albumin (BSA, lyophilized powder, ≥96%), myoglobin (from equine heart, essentially salt-free, lyophilized powder, ≥90% (SDS-PAGE)), albumin (from chicken egg white, lyophilized powder, ≥98%, agarose gel electrophoresis), urea (≥98%), trypsin (BRP grade), and thiourea (puriss. p.a., ACS reagent, ≥99.0%), were all obtained from Sigma (Zwijndrecht, The Netherlands). Human IMR90 lung fibroblast cells (ATCC CCL-186) were prepared according to what was described in ref (26). Solid-phase extraction (SPE) was performed with C18 cartridges (Supelco; 1 mL, 100 mg bed, pore size 70 Å).

### 2.2 Instrumentation

All experiments in this study were carried out using an Agilent 1290 series Infinity 2DLC system (Agilent, Waldbronn, Germany). The system comprised two binary Infinity I pumps (G4220A), one 1100 isocratic pump (G1310A), an autosampler (G4226A), a thermostated column compartment (G1316C), a valve drive (G1170A), equipped with an 8-port 2-position 2DLC valve (G4236A), and a diode-array detector (G4212A) with Agilent Max-Light cartridge cells (G4212–60008, 10 mm, detector volume = 1.0 μL). The system was controlled using Agilent OpenLAB CDS Chemstation Edition (Version 3.2 (Build 3.2.0.620)) software. The ^1^D columns were Agilent InfinityLab Poroshell 120 HPH-C18 (150 × 2.1 mm, 1.9 μm), Agilent ZORBAX SB-CN, and Agilent ZORBAX Eclipse Plus C18 (both 150 × 2.1 mm, 1.8 μm). The ^2^D column was a ZORBAX Eclipse Plus C18 (50 × 2.1 mm, 1.8 μm). In addition, an Agilent ZORBAX SB-CN (50 × 2.1 mm, 1.8 μm) and an Agilent InfinityLab Poroshell 120 HPH-C18 (50 × 2.1 mm, 1.9 μm) were used.

To perform SPAM in the 2DLC experiments, two Phenomenex SecurityGuard ULTRA C18 Cartridges (2 × 2.1 mm) were used with the corresponding Phenomenex SecurityGuard ULTRA Holders. The mass spectrometer used was Q-Exactive Plus (Thermo Scientific, Bremen, Germany).

### 2.3 Procedures

#### 2.3.1 Sample Preparation

A protein digest sample consisting of BSA or four different proteins (BSA, α-casein, myoglobin, and albumin) was used for method development. A cell lysate (human IMR90 lung fibroblast cells) was used to prove the method’s applicability. The sample preparation is based on previously described work.^27^ Details on procedures are described in Supporting Information (SI) Section S-1.

#### 2.3.2 One-Dimensional LC-HRMS

For all 1DLC experiments, the column was directly connected to an MS source. The injection volume was 2 μL, and the column thermostat was 50 °C. Mobile phase A consisted of H_2_O and mobile phase B was ACN. To both mobile phases, 0.1% FA was added with the exception of the mobile phase used for the HPH-C18 column. For the high-pH separations, A was H_2_O with 20 mM AmFm at pH 10 (adjusted using ammonium), and B was plain ACN. A full overview of the used MS settings is presented in the Supporting Information Section S-2.

When comparing 1DLC with 2DLC, 1DLC has a 150 × 2.1 mm column, a flow rate of 0.16 mL min^–1^, and a gradient from 2 to 38%B in 60 min. The exact gradient programming for all of these experiments can be found in the Supporting Information Section S-3.

#### 2.3.3 RPLC × RPLC-HRMS Method

Sample, solvent composition (of both ^1^D and ^2^D separations), column temperature, and MS settings were the same as described in the previous section. For the 2DLC experiments, the ^1^D column was 150 mm in length and the HPH-C18 for full and shifted gradients while the CN was used for parallel gradients and the ^2^D column was C18. A schematic overview of the system is depicted in Figure S1 of the Supporting Information. Two μL sample was injected for the protein mix and 15 μL for the cell lysate. In all cases where the ^1^D flow rate was 0.16 mL min^–1^, the ^1^D gradient for the HPH-C18 column was programmed in the following steps: 0–60–65–70–70.01 min and respectively 2–38–90–90–2 for the percentage of B. For the CN column, the third step was reduced from 38 to 32% B. For all methods, a modulation time of 30 s was used. For SPAM, a dilution ratio of 1:3 (water, 0.1%FA) was applied (0.48 mL min^–1^).

The full-gradient and shifted-gradient methods used the HPH-C18 column and solvents in the first dimension and the 50 mm C18 in the second dimension at a flow rate of 1.4 mL min^–1^ and a 1:1 ratio flow split prior to the MS. The full-gradient method employed a linear solvent gradient from 2 to 45% B in every modulation over the first 0.43 min, followed by 0.07 min equilibration at 2% B. The detailed shited gradient programming can be found in Section S-4.

For the parallel gradient, the 150 mm CN column was used for the ^1^D. The ^2^D gradient was programmed in the following steps: 0–60–65–70–70.01 min and 12–40–90–90–12, respectively, for the percentage of B. Contrary to the other methods, the ^2^D flow rate was set at 0.7 mL min^–1^ and therefore without the use of a flow splitter.

#### 2.3.4 MS Conditions and Data Handling

MS data were recorded by using a HESI source. A full overview of the used MS settings is presented in the Supporting Information Section S-2. Plotting of the 2DLC chromatograms and other calculations were performed using MATLAB R2024a. For the equations used to calculate several parameters, the reader is referred to Supporting Information Section S-5. MZmine version 3.90 was used for feature detection from LC and LC × LC-MS experiments.^28^ Peptide and protein identification was performed in MaxQuant (V2.1). Carbamidomethyl was used as a fixed modification, and the variable modifications were set to oxidation and acetylation. Trypsin was specified as the enzyme with a maximum of two missed cleavages. The false discovery rate (FDR) for peptide identification was set to 1%. Further details can be found in the Supporting Information Section S-6 Raw data and MaxQuant analysis results are available at https://massive.ucsd.edu/ProteoSAFe data set MSV000094598.

## 3 Results and Discussion

Here, we describe the development of a parallel-gradient LC × LC method based on RPLC separations on both dimensions and compare (in Section 3.3) it to a full and a shifted-gradient method similar to previous research.^4^

### 3.1 Screening of ^1^D Selectivities

**Figure 1:**
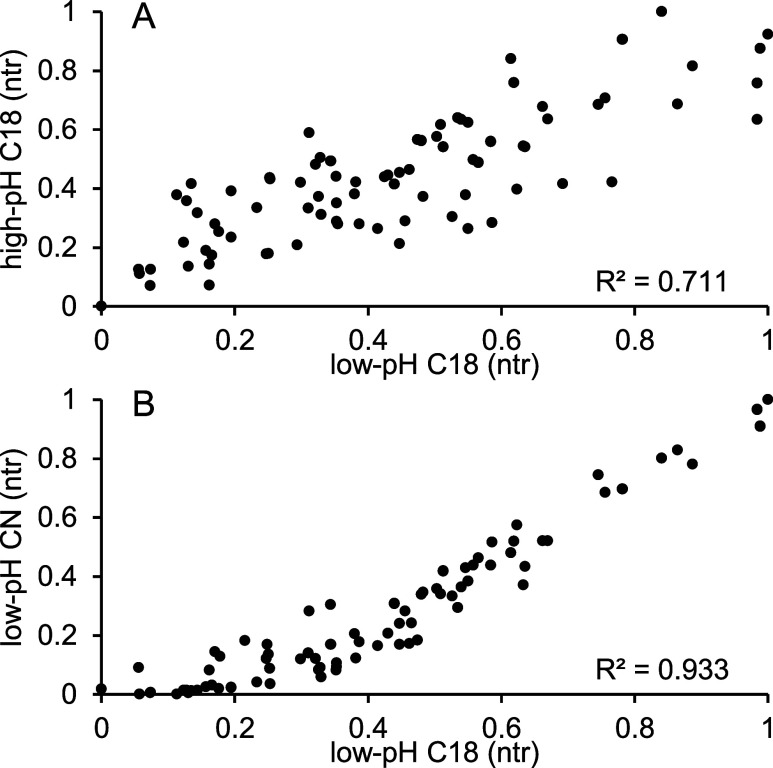
Orthogonality plots using normalized retention times (ntr) of targeted peptide features. The following comparisons are presented: C18 using 0.1% FA (*x*-axis in all subplots) vs HPH C18 using 20 mM AmFm at pH 10 (A), cyano using 0.1% FA (B).

To effectively use the 2DLC separation space in LC × LC separations using full-gradient programs, the two coupled methods should have the highest orthogonality (lowest correlation) possible. In contrast, for shifted and parallel-gradient methods, the two separation dimensions must be correlated but feature different selectivities (e.g., different elution orders of analytes).^19,20^ Therefore, to establish a parallel-RPLC × RPLC method, we initially screened several RPLC peptide separation methods (see Supporting Information Section S-7). We then selected three RPLC methods (using 150 × 2.1 mm ID columns) and measured the correlation between the LC-MS separation of a trypsin digest of a mixture of four proteins (BSA, α-casein, myoglobin, and albumin). All of the methods used ACN as an organic modifier, low-pH C18 (LPH) and cyano (CN) using 0.1% FA, and high-pH C18 (HPH) using a 20 mM AmFm at pH 10. To evaluate the results, we used the *R*^2^-value from a linear trendline of the normalized analyte retention time (ntr, calculated as (*t*_r,i_ – *t*_r,first_)/(*t*_r,last_ – *t*_r,first_)) of a specific analyte for each LC separation (Figure 1). A lower *R*^2^-value represents a lower correlation and therefore higher orthogonality of the compared selectivities. The lowest correlation (*R*^2^ = 0.711) was observed between the LPH and HPH, whereas the correlation between the LPH and CN (at the same pH) was significantly higher (*R*^2^ = 0.933). This is likely to occur due to changes in the charge of peptides between the different pH environments of HPH and LPH separations. Large differences in retention times between separation dimensions are not beneficial for parallel-gradient methods. They may lead to part of the compounds being unretained or not eluting within the modulation time (wrap-around) or having significantly wider peak widths due to high retention. Because of the high correlation but sufficient differences observed in the analyte elution order, we used the CN × LPH combination to develop a parallel-gradient RPLC × RPLC. In the case of shifted gradients, HPH × LPH was used following what was reported in a previous study.^4^

### 3.2 RPLC × RPLC Method Development: Modulation and ^1^D Method

The target of our method development was to establish a 60 min gradient time to realize analysis with medium-throughput potentials. A modulation time of 30 s was chosen for all 2DLC methods as it allows for frequent fractionation of the ^1^D and running ^2^D gradients with high gradient volumes. Previous research on 2DLC parallel gradients underlined the negative effect of injection band broadening on the method’s peak capacity when using passive modulation (i.e., sampling loops where no analyte focusing takes place).^19^ Therefore, we applied stationary-phase-assisted modulation (SPAM) as the modulation approach for the 2DLC methods. SPAM allowed diluting the ^1^D eluent, facilitating analyte focusing on trap columns before injection in ^2^D gradients, reducing band broadening between separations. We selected a 1:3 dilution ratio given the steep retention curves that peptides exhibit on C18 stationary phases (see Supporting Information Section S-7, Figure S4).

To develop ^1^D, we opted for 0.16 mL min^–1^ as the flow rate as a result of the Van Deemter curve analysis of the CN column (see Supporting Information Section S-7, Figure S5). We then tested linear gradients for the CN and HPH separations. The protein mixture digest was used as the model sample for method development and evaluation purposes. The gradient slope was adjusted such that the peaks elute within 60 min, to spread the analytes as well as possible within the gradient time. The methods we selected for the HPH and CN columns used gradients from 2 to 38% and 2–32% ACN. Feature peak detection was performed on 146 masses (list and description are reported in Supporting Information files) and we obtained average full widths at half height (FWHM) of 0.121 min (HPH) and 0.172 min (CN) and a corresponding peak capacity of about 292 and 206 (*t*_g_ = 60 min). In addition, an LPH method, which will be used as a state-of-the-art 1DLC reference, was developed with a gradient from 2 to 38% B, resulting in peaks with an average FWHM of 0.120 min and a peak capacity of about 295.

#### 3.2.1 Full- and Shifted-Gradient RPLC × RPLC-HRMS Methods

Full- and shifted-gradient methods were developed following principles discussed in a recent 2DLC literature,^4,14^ coupling an HPH ^1^D with LPH ^2^D separation. We used high ^2^D flow rates (1.4 mL min^–1^) to increase the gradient volume and shorten the re-equilibration time for each ^2^D separation. However, the maximum flow rate allowed from our ESI source was 0.7 mL min^–1^ and therefore we applied postcolumn 1:1 flow-split.

**Figure 2:**
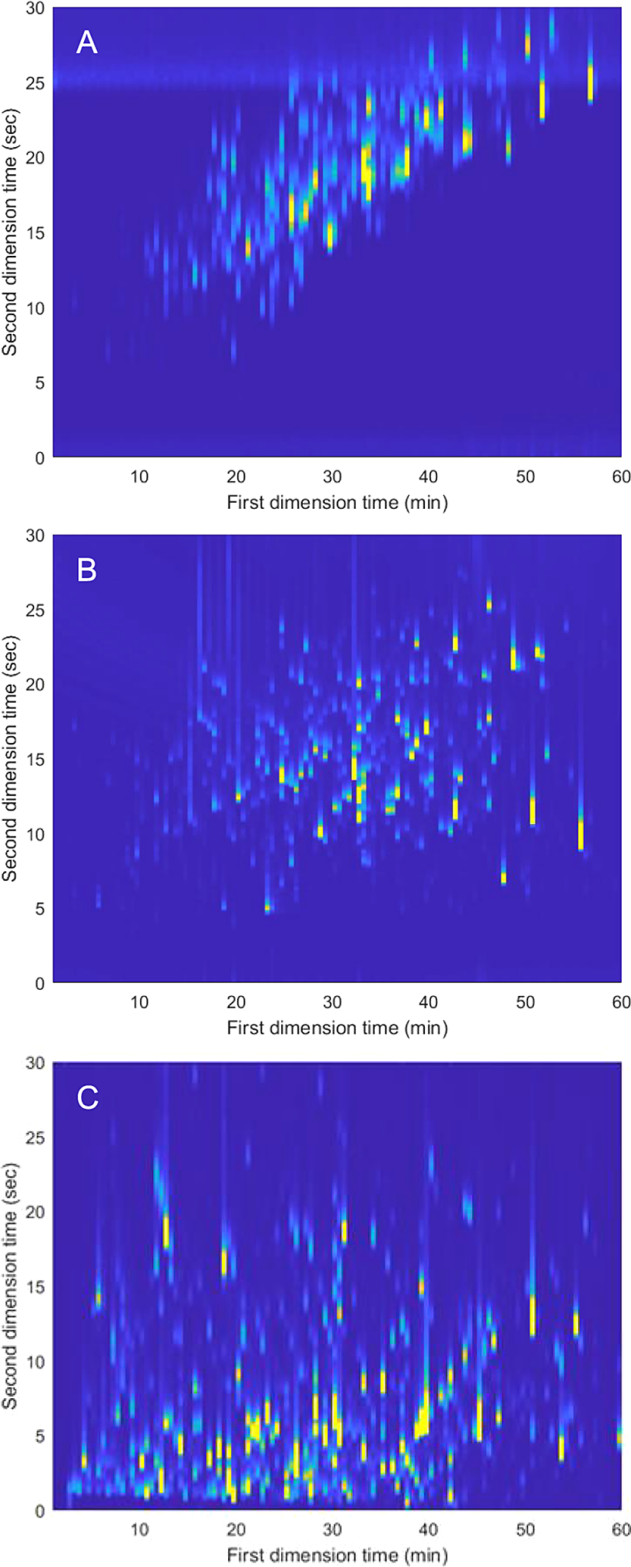
Two-dimensional LC plots of the BPC obtained from the protein-mixture digest sample using different gradient assemblies: full gradient (A), shifted gradient (B), and parallel gradient (C). In all plots, the intensity is represented by color and scaled to a relative intensity such that all chromatograms appear equally visible despite absolute differences in peak heights. It should be noted for ease of visibility, the ^2^D times in Figure A, B have been shifted by 0.07 min and C by 0.24 min to account for dead time.

Figure 2A displays the results from the full-gradient RPLC × RPLC separation in the analysis of the protein mixture digest. This method presents a high correlation and, therefore, low 2DLC space utilization, as can be observed by the clustering of the peaks. The results of the full-gradient measurements were used to design a shifted-gradient program, allowing us to extrapolate the upper and lower boundaries and times of the ^2^D shifted gradient. Briefly, we maintained the lower boundary of 6% B in the ^2^D until 20 min; this was then increased linearly to 35% B until 60 min. The upper ^2^D %B boundary started at 30% B and then increased linearly to 45% at 20 min and kept constant until the end of the run. Figure 2B presents the result of the analysis of the protein mixture digested by the shifted HPC × LPH method. The shifted-gradient programming significantly increased the utilization of the 2DLC space.

#### 3.2.2 Parallel Gradient RPLC × RPLC-HRMS Method

To develop the parallel gradient, we selected the CN × LPH combination following what was described in Section 3.1. The ^1^D gradient method is described in the next Section 3.2. The ^1^D flow rate and modulation parameters were identical to those of the other methods. The ^2^D flow rate was 0.7 mL min^–1^ allowing for splittless MS coupling (vs 1.4 mL min^–1^ of full- and shifted gradients). This was possible because in parallel-gradient RPLC × RPLC, there is no need for equilibration time between ^2^D separations and it is not needed to deliver, in a short time, a gradient of several ^2^D column volumes. This resulted in the inherent advantage of a reduced 2DLC dilution factor and avoiding flow splitting. However, broader ^2^D peaks are expected from this gradient design due to the lower flow rate and shallow gradient elution conditions. Conversely, filling up the entire space without the need for column re-equilibration may increase the use of the separation space (surface coverage) and, therefore, the effective peak capacity.

For the ^2^D separation, a continuous gradient running parallel to the ^1^D gradient was developed. The ^2^D gradient used a higher modifier percentage than the ^1^D gradient, as higher retention was present in the LPH with respect to the CN method. Different offsets and slopes were tested to increase the spread of ^2^D peaks over the modulation time while avoiding excessive retention to minimize peak broadening. Finally, a ^2^D gradient program running from 12 to 40% B was chosen. Figure 2C displays the obtained 2DLC chromatogram of the analysis of the protein mixture digest.

### 3.3 Comparison of the 2DLC Separation Methods

In this section, the performance of shifted and parallel-gradient methods will be compared in terms of (i) separation metrics, (ii) run-to-run repeatability and sensitivity, and (iii) data-dependent MS/MS protein identification analysis of a cell protein digest. In addition, in (iii), we will discuss the importance of the modulation time in parallel gradients to increase protein and peptide metrics. Key data used for comparison are summarized in Table 1.

**Table 1:** Comparison of 1D- and 2DLC Methods for the Analysis of Protein Digests Extracting Average Data from Detected Features in Terms of Peak Width, Height, and Area^a^

**method**	**1D C18**	**full**	**shifted**	**parallel**
FWHM (min)	0.1212	0.0161	0.0129	0.0181
height (counts)	2.23 × 10^07^	4.63 × 10^06^	1.26 × 10^06^	8.93 × 10^06^
dilution factor (2D)	N/A	288	232	162
area (counts min)	1.51 × 10^08^	6.69 × 10^08^	9.02 × 10^07^	3.92 × 10^08^
SC	N/A	0.2613^b^	0.6226	0.7439
*n*’_c_	292	263^c^	779^c^	757^c^
*t*_r_rep. (s) (*n* = 4)	N/A	N/A	0.2947	0.2877

a In addition, data on surface coverage, effective peak capacity, and retention time repeatability (*n* = 4) are reported.

b Only 100 features were used for the full-gradient, as opposed to 300 for the other methods, as a significant peak overlap was observed.

c Results obtained correcting for surface coverage (SC) and undersampling factor.

#### 3.3.1 Effective Peak Capacities of Shifted- and Parallel Gradients

We evaluated the separation performance of the methods by calculating the effective peak capacity. This was obtained by combining the results of ^1^D and ^2^D peak capacity (^1^D peak capacity data are discussed in Section 3.2), undersampling factor, and 2DLC surface coverage analysis, following what was described in previous studies.^29,30^

The ^2^D peak capacity was calculated from the peak width from the feature detection analysis of 73 unique features having the highest peak height in shifted- and parallel-gradient methods. Broader peak widths (FWHM 0.0181 parallel vs 0.0129 min shifted) were observed with parallel gradients (see Figure S7). These results can be explained by the lower ^2^D flow rate and the more limited gradient peak compression effects. Moreover, in parallel gradients, analytes may have long retention times and possibly not elute within one modulation (wrap-around). The ^2^D peak capacities of roughly 16 (parallel) and 19.5 (shifted) per modulation were obtained.

To calculate the extent to which the ^1^D peak capacity was kept due to the sampling frequency of our 2DLC methods we calculated the undersampling factor.^30^ This factor was higher for shifted gradients (4.56) with respect to parallel gradients (3.28) as the HPH separation had a higher peak capacity with respect to the CN.

Next, we investigated the use of the 2DLC separation space surface using the convex hull method.^4,20,31^ This algorithm connects the outermost data points in a space with straight lines and computes the area of its inner surface. This surface area is then divided by the total available separation space to obtain a value between zero and one, where one represents full surface coverage (SC). In our study, to get the most fair comparison between the different gradient approaches, the complete 2D time was considered in all cases, and only the ^1^D dead time (2 min) was omitted. Therefore, the total available space for all chromatograms was 58 min in the ^1^D and 30 s in the ^2^D. The SC was calculated using the peak tops of the 300 most abundant peaks (see Supporting Information Section S-7, Figure S6). The full-gradient method presented the lowest surface coverage with a value of about 0.26, shifted gradient 0.62, and parallel gradient the highest with 0.74. These results highlight the fraction of the 2D separation space that was unused (74, 38, and 26%, respectively) and therefore in which the MS detector was not analyzing analyte-related *m*/*z* features. The application of shifted gradients clearly increased the usage of separation space. However, in each modulation, the first 5 s were needed for column equilibration (16% of the total ^2^D separation) and therefore not used for analysis. In the parallel gradient method (Figure 2C), as no equilibration time was needed between runs, the analytes eluted through almost the entire ^2^D time. This resulted in a higher surface coverage, roughly 19% more than that obtained using a shifted-gradient program.

Finally, we calculated the effective peak capacity from the parameter described above (see Supporting Information Section S-5 for details), obtaining a value of 779 for the shifted gradient and 757 for the parallel gradient. The two methods provide similar separation performances, with the parallel gradient allowing for higher utilization of the 2DLC separation space (surface coverage) and the shifted gradients enabling sharper ^2^D peaks. Parallel and shifted gradients outperformed the full-gradient method and the 1D LPH method (peak capacities of 288 and 295 respectively).

#### 3.3.2 Run-to-Run Repeatability and RPLC × RPLC-HRMS Sensitivity

To achieve widespread implementation of LC × LC methods for routine use, run-to-run repeatability is a crucial factor. To assess this, the shifted and parallel-gradient methods were subjected to four consecutive injections of the protein digest mixture, and the variation in ^2^D elution times between four runs was evaluated. Common features presented in all four measurements that eluted within one modulation were selected using the batch-pairing algorithm.^32^ For the shifted-gradient method, the average standard deviation over the ^2^D retention times was 0.2947 s, while for the parallel-gradient method, it was 0.2877 s. The distributions of the average retention time variation (*n* = 4) for all these features are displayed in Figure S8 in Supporting Information Section S-7. We concluded that parallel- and shifted-gradient methods present similar deviations in ^2^D retention times and can be considered sufficiently repeatable as both averages were below 0.3 s, which was only several data points at the MS acquisition rate (between about 2 and 10 Hz).

Next, we investigated the difference in sensitivity of the methods by applying feature detection and extracting peak area and heights. We observed a clear gain in sensitivity when using the parallel-gradient method, with about eight times higher average area (8.94 × 10^8^ vs 1.26 × 10^8^) and four times higher average peak height (3.92 × 10^8^ and 9.22 × 10^7^). The difference observed was likely a result of the higher dilution factor in the shifted-gradients ^2^D separation where the flow rate was double the one of the parallel gradient (1.4 vs 0.7 mL min^–1^). This was reflected in the higher calculated dilution factor (232 vs 162).

#### 3.3.3 RPLC × RPLC-MS/MS of a Cell Lysate Digest

Finally, we tested if the increased separation power of the RPLC × RPLC methods developed yields higher protein identifications in the analysis of complex proteomics samples. To benchmark the methods’ performance, we applied the parallel, shifted gradients, and 1DLC LPH method to analyze the same amount of a complex protein digest (cell lysate (CL) of Human IMR90 lung fibroblast cells) in the same analysis time. This sample was selected as a representative sample for proteomics application with thousands of proteins present, which were subsequently digested.

**Figure 3:**
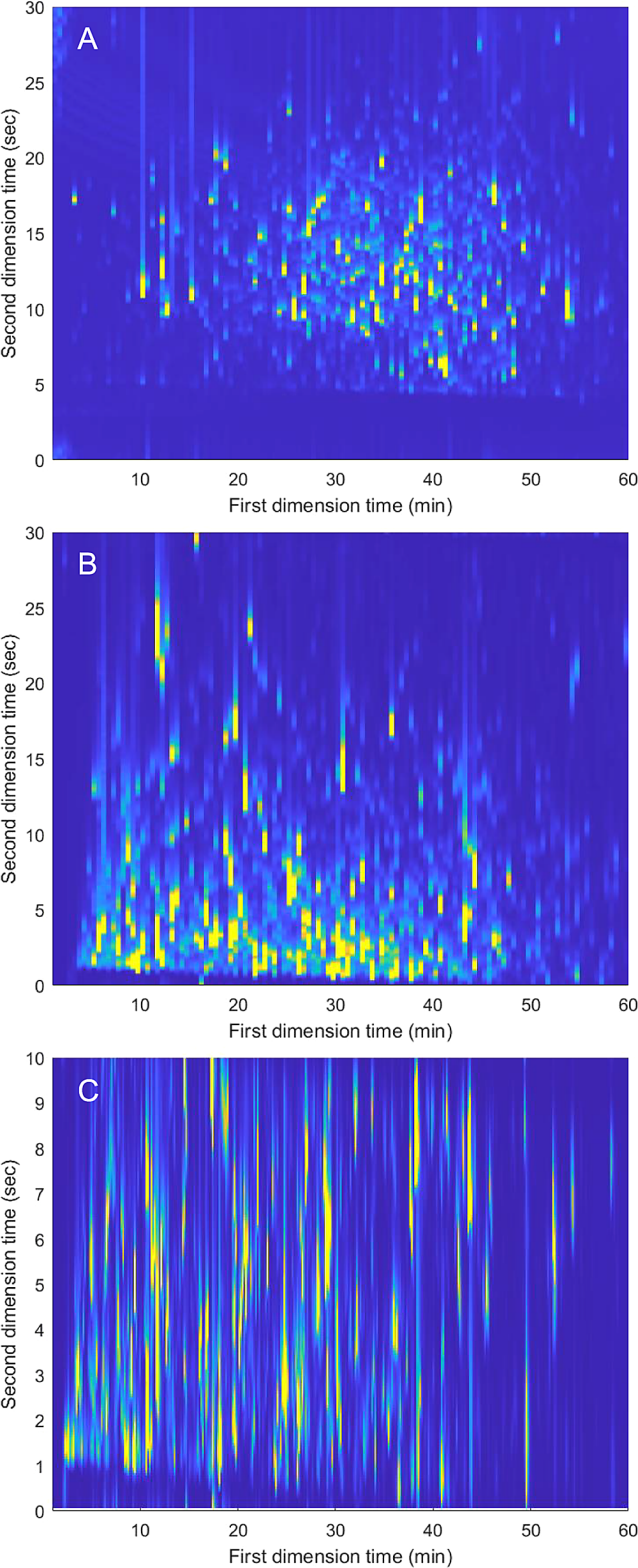
Base-peak RPLC × RPLC-MS/MS chromatograms of a cell lystate of human IMR90 lung fibroblast cells using the shifted-gradient method (A) and the parallel-gradient method using 30 s modulations (B) and 10 s modulations (C). 1DLC analysis view of the data can be found in Figure S11.

Figure 3 displays the 2DLC chromatograms for shifted and parallel-gradient method analysis of the CL. In both methods, significantly more peaks were visible than in the protein mixture digest used for method development. However, surface coverage, and peak width results were similar. A top 6 MS/MS data-dependent analysis was used to identify peptide sequences and infer the presence of proteins. Table 2 summarizes the results of the MS protein analysis (Supporting Information Section S-7, Table S9 for full details). We observed important differences in MS/MS results and peptide and protein identifications between the data sets. In particular, we observed in the 1D LPH method a higher MS/MS ratio over that of full MS scans. This indicated that throughout the analysis time five or more *m*/*z* features (not in the exclusion list dynamically changed every 20 s) and had intensity over the set threshold. In comparison, shifted and parallel gradients had lower ratios of about three. In the 1D LPH experiments, the peptides continuously eluted from the column with no gaps of analyte elution in the total ion chromatogram. This was not the case for 2DLC measurements where gaps where no analytes eluted are present; therefore, a lower number of MS/MS events took place. Moreover, 2DLC methods, despite significantly increasing the peak capacity, may present lower sensitivity as a result of the higher flow rates used in the ^2^D separation and fractionation of ^1^D peaks into multiple ^2^D separations. This is true in particular for shifted gradients where lower average MS intensity with respect to the 1D LPH method (about 4 times less) was observed. We suggest that this is the reason why the gains in separation obtained by both shifted and parallel gradients do not offer increased identification. In particular, shifted gradients perform significantly worse than the 1D LPH method, identifying about 80 and 45% fewer peptides and proteins. Instead, the parallel-gradient methods gave results similar to the 1D LPH method with a comparable number of proteins identified despite a lower number of peptides identified (33% less), indicating that the 2DLC method may reduce the number of peptides from the same protein.

**Table 2:** Number of MS and MS/MS Events Observed in the 1D- and 2DLC-HRMS Dataset in the Elution Area between 2 and 60 min and the Related Peptide and Protein Identifications

**method**	MS/MS	**ratio MS/MS to MS**	**average MS intensity**	**peptide IDs**	**protein IDs**
1D-C18 1	25,568	5.27	9.43 × 10^06^	5858	1539
1D-C18 2	26,198	5.49	1.10 × 10^07^	6275	1554
shift 1	19,312	3.29	2.71 × 10^06^	2670	977
shift 2	19,157	3.25	2.17 × 10^06^	2565	967
parallel 30 s 1	20,780	3.60	1.43 × 10^07^	4142	1539
parallel 30 s 2	21,106	3.69	1.42 × 10^07^	3774	1554
parallel 20 s 1	24,425	4.73	1.23 × 10^07^	5420	1786
parallel 20 s 2	24,484	4.77	1.15 × 10^07^	5086	1730
parallel 10 s 1	26,262	5.37	1.44 × 10^07^	7177	1994
parallel 10 s 2	26,170	5.36	1.47 × 10^07^	7144	1989

Following these results, we further developed the parallel-gradient method to improve its LC-MS/MS performance. To achieve this, we decreased the modulation time to 20 and 10 s aiming to use wrap-around effects (analytes eluting after one modulation) to occupy the less-used ^2^D space in the second half of the initial modulation (Supporting Information Section S-7, Figures S9–S11) and fill the gaps between modulations. However, this approach will (i) reduce the peak capacity of the method, as the lower modulation time will result in a lower peak capacity per ^2^D and (ii) will further increase the dilution of the method as ^1^D peaks will be fractionated in more ^2^D separations (thus potentially reduce peak heights).

In our experiments, increasing the modulation frequency in parallel gradients significantly increased the number of MS/MS events, reaching values similar to the one of 1D LPH (ratio MS/MS to MS of 5), demonstrating that distributing the analytes within the ^2^D separation and reducing the gap analyte elution gaps between modulations has a significant effect in the analysis of highly complicated samples. The best results in terms of protein analysis from 2DLC experiments were achieved using 10 s modulations with an increase of peptides (12%) and proteins (22%) identified with respect to 1D LPH. Interestingly, these results were achieved despite reducing the calculated 2DLC effective peak capacity (706 and 564 using 20 and 10 s modulation times).

## 4 Conclusions

This study compared the use of shifted and parallel-gradient designs for the second dimension in correlated LC × LC separations. Shifted gradients can achieve the highest effective peak capacities and narrowest peak widths. They also achieve significantly better surface coverage than the conventional full-gradient approaches. However, to achieve high peak capacity ^2^D separations, high flow rates have to be used, reducing the MS sensitivity. Moreover, part of the separation space is solely used for ^2^D column re-equilibration, introducing gaps in the MS/MS analysis.

Parallel gradients provide lower effective peak capacity but have higher 2DLC surface coverage and sensitivity. Furthermore, this approach had a lower organic solvent (ACN) consumption (35.7 and 19.5 mL per run for shifted- and parallel gradient, respectively). Most importantly, in analyzing highly complex protein digests by MS/MS, parallel gradients obtained significantly higher protein identification numbers than those obtained by the shifted-gradient method. Moreover, reducing the modulation time (here to 10 s) allowed to exploit wrap-around effects, allowing to more evenly distribute the analytes within each modulation.

In future studies, it may be valuable to consider these shorter modulation times for parallel-gradient designs in RPLC × RPLC. Moreover, MS instrumentation with higher MS and MS/MS frequencies and data acquisition strategies such as MS/MS data-independent analysis may be able to take even greater advantage of the extra separation power offered by LC × LC compared with that of 1D separations. Automated method development may aid in simplifying the design of both shifted and parallel-gradient designs and may further improve the overall performance of such methods. Lastly, it should be repeated that striving for maximal peak capacity or surface coverage will not always contribute to the goal of the analytical method, and therefore, the metric used to describe the performance of the separation should carefully be selected.

## Supporting Information Available

Details regarding the sample preparation, instrumental settings, data-processing protocols, figures displaying additional experimental data, and CRediT authorship contribution statement (PDF)
Feature list (XLSX)
Feature list and 2DLC metrics (XLSX)
