# Supplementary material for: Comprehensive Two-Dimensional Liquid Chromatography–High-Resolution Mass Spectrometry for Complex Protein Digest Analysis Using Parallel Gradients
Source: Anal Chem. 2024 May 17;96(22):9294–301. doi: 10.1021/acs.analchem.4c02172 (PMC11154668; doi:10.1021/acs.analchem.4c02172)
Supplement: Supplementary file 1 — ac4c02172_si_001.pdf [file ac4c02172_si_001.pdf]

# SUPPORTING INFORMATION

## Comprehensive two-dimensional liquid chromatography – high-resolution mass spectrometry for complex protein digest analysis using parallel gradients

Rick S. van den Hurk [a,b], Bart Lagerwaard [a,b], Nathan J. Terlouw [a,b], Mingzhe Sun [a,b], Job J. Tieleman [a,b], Anniek X. Verstegen [a,b], Saer Samanipour [a,b], Bob W.J. Pirok [a,b] , Andrea F.G. Gargano [a,b*]

[a] Analytical Chemistry Group, Van 't Hoff Institute for Molecular Sciences, University of Amsterdam, The Netherlands

[b] Centre for Analytical Sciences Amsterdam (CASA), the Netherlands

**CRediT authorship contribution statement**

**Rick S. van den Hurk:** Conceptualization, Methodology, Investigation, Formal Analysis, Writing – Original draft, Visualization. **Bart Lagerwaard:** Conceptualization, Methodology, Investigation, Formal Analysis, Visualization. **Mingzhe Sun:** Investigation, Formal Analysis**. Nathan J. Terlouw:** Investigation, Formal Analysis**. Job Tieleman:** Investigation, Formal Analysis**. Anniek Verstegen:** Investigation, Formal Analysis. **Bob W.J. Pirok:** Writing – review & editing, Methodology, Project administration, Supervision, Resources. **Saer Samanipour:** Writing – review & editing, Resources. **Andrea F.G. Gargano:** Conceptualization, Methodology, Investigation, Writing – review & editing, Project administration, Supervision, Resources.

* Corresponding author

Andrea F.G. Gargano a.gargano@uva.nl

## Contents

S-1 Sample preparation ........ 3

S-2 Instrumental settings used for the MS ........ 4

S-3 Gradient programs used for 1D experiments ........ 5

S-4 Additional details regarding the LC×LC experiments ........ 6

S-5 Equations used for calculating separation parameters ........ 8

S-6 Details for various MS data processing protocols ........ 10

S-7 Additional experimental data ........ 12

References ........ 16

## S-1 Sample preparation

For the digestion, 10 mg of the protein stock was weighted in and added to 1 mL of 6 M urea in a 1.5mL Eppendorf tube. From a solution of 30 mg/mL DTT (reducing agent) in 25 mM ammonium bicarbonate solution, 5 µL was added and vortexed. The mixture was left to reduce for 1 hour at 37 °C in a mixing heating block. From a 36 mg/mL IAA (alkylating agent) in 25 mM ammonium bicarbonate solution, 5 µL was added and left for 1 hour at room temperature in aluminum foil. From the reducing agent, 5 µL was added in combination with 900 µL 25 mM ammonium bicarbonate. From a 1mg/mL trypsin stock, 33.3 µL was added and left 24 hours for digestion.

The next day, 40 µL of 10% TFA in water was added to acidify the sample. The protein digests were desalted using C18 solid-phase extraction (SPE) cartridges. The SPE cartridges were inserted into a vacuum manifold connected to a vacuum pump. The cartridges were activated with ACN and equilibrated with 0.1% TFA in water. After loading the sample, the cartridges were drained and washed with the 0.1% TFA solution. The protein digest was extracted from the cartridge with 500 µL 75% ACN/0.1% TFA in water and eluted into an 1.5 mL Eppendorf tube. The solutions were concentrated to a pellet using a freeze drier. After 24 hours the sample was taken out of the freeze drier and stored in a freezer (-20 °C). Just before the experiments, the sample was dissolved in 500 µL 98% $H_2O$/2% ACN with 0.1% TFA.

The 4 proteins used as reference were combined after the desalting procedure. For the cell lysate protein precipitation before digestion was performed. The proteins were precipitated with cold acetone (-20C) and centrifuged for 10 minutes at 13,000 – 15,000 x g. The acetone was then removed and the pellet was suspended in 100 µL of 6 M urea solution. The proteins were then digested according to the digestion protocol.

## S-2 Instrumental settings used for the MS

**Table S1:** MS settings used for full-scan mode.

| Full MS properties | MS |
|---|---|
| Scan range | 300.0 to 1.600 m/z |
| Fragmentation | None |
| Resolution | 35.000 (1D analysis) / 70.000 (for MS/MS) |
| Polarity | Positive |
| Microscans | 1 |
| Lock masses | Off |
| AGC target | 3e6 |
| Maximum inject time | 100 ms (1D analysis) / 50 ms (for MS/MS) |

**Table S2:** Ms settings used for MS/MS mode.

| MS/MS properties | dd-$MS^2$ |
|---|---|
| Resolution | 17.500 |
| AGC target | 1e5 |
| Maximum inject time | 40 ms |
| Loop count | 6 |
| TopN | 6 |
| Isolation window | 1.8 m/z |
| Fixed first mass | 110.0 m/z |
| (N)CE / stepped (N)CE | nce : 27 |
| Minimum AGC target | 7.10e2 |
| Intensity threshold | 1.8e4 |
| Apex trigger | - |
| Charge exclusion | Unassigned, 1, 6-8, >8 |
| Peptide match | Preferred |
| Exclude isotopes | On |
| Dynamic exclusion | 20.0 s |

**Table S3:** ESI source conditions used per flow rate of the LC effluent.

| HESI source parameters | Low flow | 0.7 mL $min^{-1}$ |
|---|---|---|
| Sheath gas flow rate | 35 | 60 |
| Aux gas flow rate | 10 | 20 |
| Sweep gas flow rate | 0 | 0 |
| Spray voltage (kV) | 2.50 | 3.50 |
| Spray current (µA) | - | - |
| Capillary temp. (°C) | 350 | 380 |
| S-lens RF level | 50.0 | 50.0 |
| Aux gas heater temp (°C) | 250 | 300 |

## S-3 Gradient programs used for 1D experiments

**Table S4:** Gradient program used for exploring selectivities on 50 mm columns.

| Time (min) | B (%) |
|---|---|
| 0 | 2 |
| 7.2 | 50 |
| 8 | 50 |
| 8.01 | 2 |

**Table S5:** Gradient program used for 1D-LC for comparison to 2D and for $^{1}$D peak capacities.

| Time (min) | B (%) |
|---|---|
| 0 | 2 |
| 60 | 38 |
| 65 | 90 |
| 70 | 90 |
| 70.01 | 2 |

## S-4 Additional details regarding the LC×LC experiments

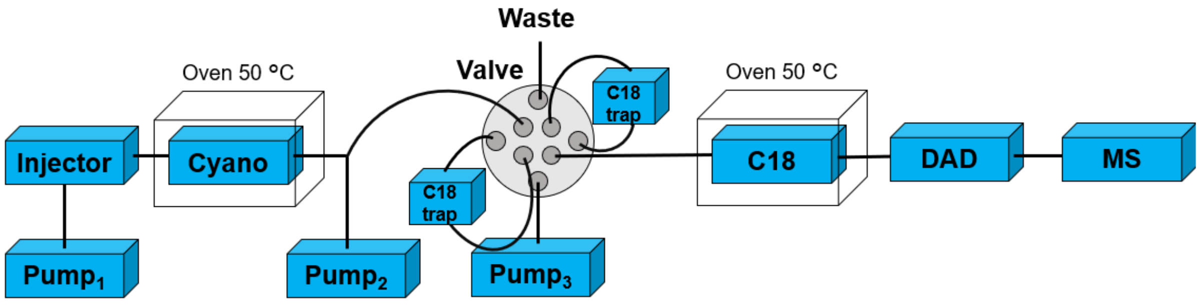


**Figure S1:** Schematic overview of the CN×RPLC stationary-phase-assisted modulation setup.

**Table S6:** Gradient programs (60-minute) used in the RPLC×RPLC methods. The HPH and CN gradients were the first-dimension gradient programs and the C18 was the second-dimension gradient of the parallel gradient method. Below the table is reported a screenshot that shows how parallel-gradient methods can be applied to the agilent software.

| Time (min) | $^1$D HPH B (%) | $^1$D CN B (%) | $^2$D C18 B (%) |
|---|---|---|---|
| 0 | 2 | 2 | 12 |
| 60 | 38 | 32 | 38 |
| 65 | 90 | 90 | 90 |
| 70 | 90 | 90 | 90 |
| 70.01 | 2 | 2 | 2 |

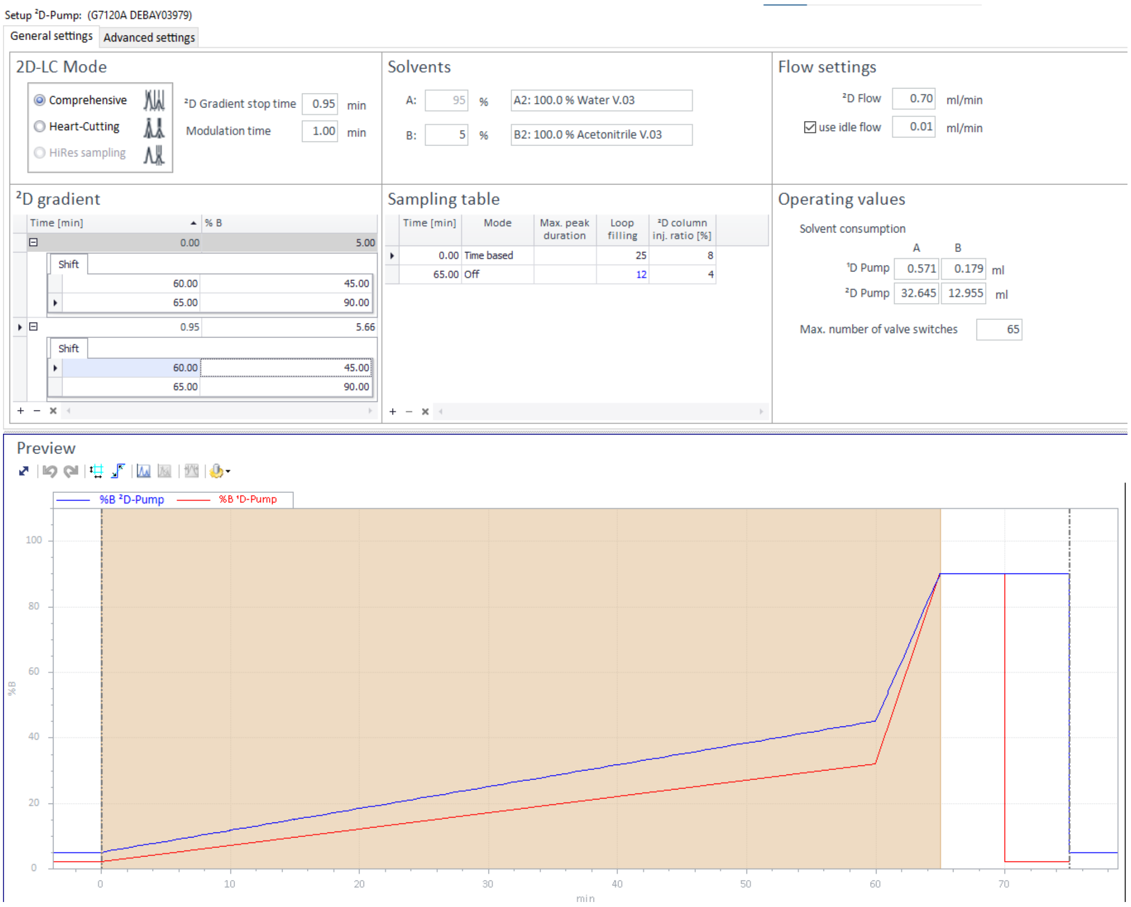

**Table S7:** Gradient programs (0.5-minute) used for $^{2}$D separation in the LC×LC methods. The shifted-gradient method contain sub tables referring to the programming of the lower and upper boundaries of the shift.

<table>
<tr><th>Time (min)</th><th>Full B (%)</th><th>Shifted B (%)</th></tr>
<tr><td>0</td><td>2</td><td>6<br><table><tr><th>t (min)</th><th>B (%)</th></tr><tr><td>0</td><td>6</td></tr><tr><td>20</td><td>6</td></tr><tr><td>60</td><td>35</td></tr></table></td></tr>
<tr><td>0.43</td><td>45</td><td>30<br><table><tr><th>t (min)</th><th>B (%)</th></tr><tr><td>0</td><td>30</td></tr><tr><td>20</td><td>45</td></tr><tr><td>60</td><td>45</td></tr></table></td></tr>
</table>

## S-5 Equations used for calculating separation parameters

This section describes the equations used for the calculation of separation parameters such as the peak capacity, effective peak capacity [1,2], and surface coverage. The peak capacity ($n_c$) of a single separation dimension may be calculated using Equation 1. Where $t_g$ is the gradient time used and $\sigma$ is the average sigma of the peaks. Sigma can in turn be estimated using the full width at half maximum (FWHM) and divide it by 2.35.

$$n_c = 1 + \frac{t_g}{4\sigma} \quad (1)$$

The under-sampling factor ($\beta$) can be calculated using Equation 2. In this equation, the modulation time ($t_{mod}$) is required as well as the first-dimension average peak sigma ($\sigma_{1D}$).

$$\beta = \sqrt{1 + 0.21\left(\frac{t_{mod}}{\sigma_{1D}}\right)^2} \quad (2)$$

The effective peak capacity ($n'_{c,2D}$) of an LC×LC system is calculated using Equation 3. In this equation, the individual peak capacities of the first ($n_{c1}$) and second dimension ($n_{c1}$) are used. In addition, the under-sampling factor ($\beta$) is used to correct for excessive $n_{c1}$. The final parameter is the surface coverage ($S_{cov}$).

$$n'_{c,2D} = n_{c1} \times n_{c2} \times \frac{S_{cov}}{\beta} \quad (3)$$

The Surface coverage can be calculated in many ways. In this work, the sake of calculating the effective peak capacity, the Convex Hull approach was selected as metric for determining surface coverage [3]. The Convex Hull algorithm identifies the outermost point in a space and computes linear lines between them such that all datapoints fall within the lines. The next step is to calculate the area ($area_{CH}$) within the Convex Hull points. To obtain the surface coverage ($S_{cov}$), Equation 4 can be used where ($area_{tot}$) is the total available area. However, within this approach, the user has to define $area_{tot}$. An example is given below using Figure S2. In this example, the output of the Convex Hull algorithm is an $area_{CH}$ of approximately 1083. If all space is considered, $area_{tot} = 60 \times 30 = 1800$. However, one could opt to ignore the dead time of the first dimension ($area_{tot} = 58 \times 30 = 1740$). Some also argue to additionally ignore the $^2$D equilibration time ($area_{tot} = 58 \times 25 = 1450$). These options respectively result in surface coverages of 0.602, 0.622, and 0.747. In our study, the $^1$D dead time is ignored but the $^2$D is considered in its entirety.

$$S_{cov} = \frac{area_{CH}}{area_{tot}} \quad (4)$$

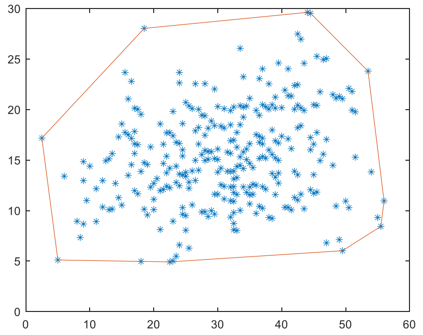


**Figure S2:** Example of Convex Hull computation of the shifted-gradient results. The peak tops (blue) are used to compute the Convex Hull boundary (orange) within the available space. The x-axis represents the $^1$D time (min) and the y-axis the $^2$D time (s).

With respect to the dilution factor (DF), the model proposed by Vivo-Truyols *et al.* [4] and later adapted by Gargano *et al.* [5] was used following Equation 5.

$$DF = \sqrt{2\pi}\frac{\sigma_{peak}F}{V_i} \qquad (5)$$

Where $\sigma_{peak}$ is the average peak width obtained as FWHM divided by 2.35, $F$ is the flow rate and $V_i$ is the injection volume. The DF of the LC×LC method ($^{2D}$DF) was calculated using Equation 6.

$$^{2D}DF = M \times \, ^{2}DF \qquad (6)$$

In this equation, $M$ is the number of modulations.

## S-6 Details about MS data processing

Mzmine was used to determine the average peakwidth of m/z features. To extract the masses from datasets, Exact Mass was used as mass detector, with noise level 5 E4, detect isotope signal below the noise level. To extract the features from 1D runs the ADAP chromatogram builder was used. 4 consecutive scans, 1E4 for the minimum consecutive scans, 5E5 for the min absolute height. Mz tollerance 0.0020 or 10 ppm

Targeted feature detection was performed on about 146 m/z features (excel table SI-1D) obtained from feature detections on CN and HPC18 runs. These were selected by restricting the search between 5 and 55 min and sorting the features on the basis of their height. Of the features detected in both datasets, the 73 unique features with the highest height were selected in each datasets. The same feature and approach was used for the analysis of the protein mix via LPH, full, shifted, and parallel 2DLC-HRMS data.

**Table S8:** Targeted feature list of 31 peptides of a BSA digest in the selectivity screening experiment in section S-7.

| Peptide | m/z | Charge |
|---|---|---|
| QRLRCASIQK | 601.757 | 2+ |
| TVMENFVAF | 537.261 | 2+ |
| CASIQKFGER | 569.756 | 2+ |
| YICDNQDTISSK | 722.331 | 2+ |
| LCVLHEK | 449.743 | 2+ |
| SLHTLFGDELCK | 473.906 | 3+ |
| FKDLGEEHFK | 417.214 | 3+ |
| LVTDLTK | 395.239 | 2+ |
| AEFVEVTK | 461.745 | 2+ |
| DLGEEHFK | 487.747 | 2+ |
| QEPERNECF | 604.772 | 2+ |
| STVFDKLK | 469.276 | 2+ |
| HLVDEPQNLIK | 653.368 | 2+ |
| KVPQVSTPTLVEVSR | 547.322 | 3+ |
| LVVSTQTALA | 501.289 | 2+ |
| RHPEYAVSVLLR | 480.605 | 3+ |
| RHPEYAVSVLLR | 720.407 | 2+ |
| RPCFSALTPDETYVPK | 627.981 | 3+ |
| RPCFSALTPDETYVPK | 941.471 | 2+ |
| KQTALVELLK | 571.864 | 2+ |
| LVNELTEFAK | 582.316 | 2+ |
| SLHTLFGDELCK | 710.358 | 2+ |
| AFDEKLF | 435.197 | 2+ |
| YLYEIAR | 464.255 | 2+ |
| VASLRETYGDMADCCEK | 630.984 | 3+ |
| QTALVELLK | 507.815 | 2+ |
| LGEYGFQNALIVR | 740.406 | 2+ |
| HADICTLPDTEK | 700.345 | 2+ |
| LQQCPFDEHVK | 700.843 | 2+ |
| LQQCPFDEHVK | 467.565 | 3+ |
| SQYLQQCPFDEHVK | 593.598 | 3+ |

## S-7 Additional experimental data

To allow the comparison of columns with varying dimensions, the linear velocity of the mobile phase was kept constant, and gradients were programmed such that the ratio of gradient time ($t_g$) and dead time ($t_0$) remained constant. All measurements were carried out in triplicates. Features are reported in Table S-8.

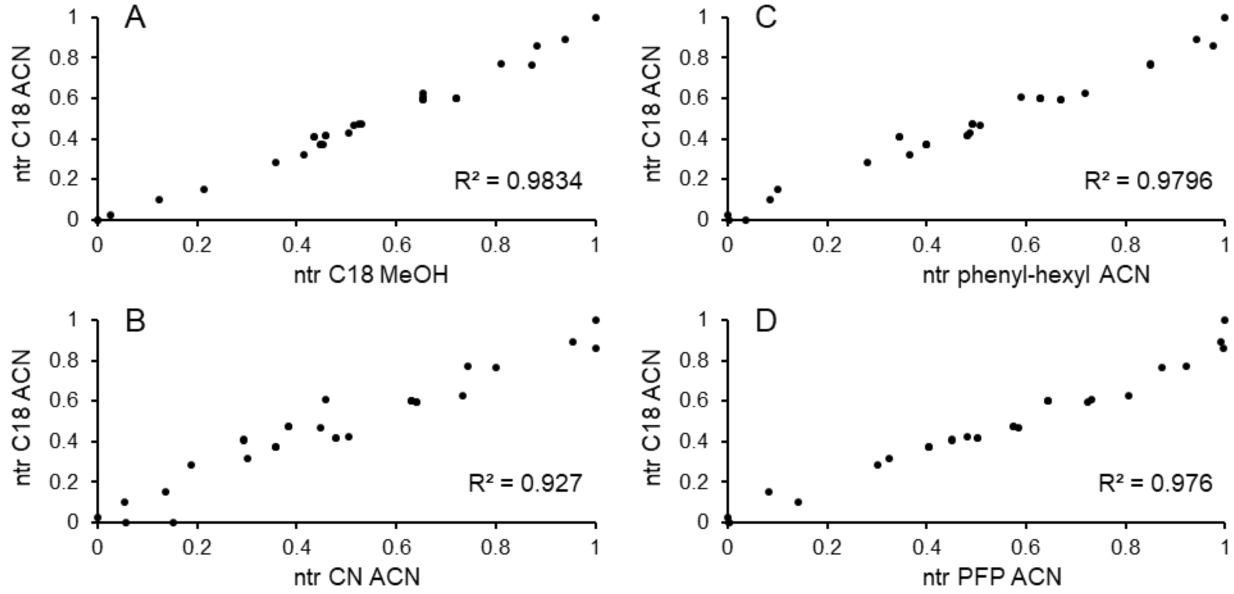


**Figure S3:** Orthogonality plots using normalized retention times (ntr) of a subset of targeted peptide features. The following four comparisons are presented: C18 using ACN modifier (y-axis in all subplots) vs C18 MeOH (A), cyano (B), phenyl-hexyl (C), and PFP (D).

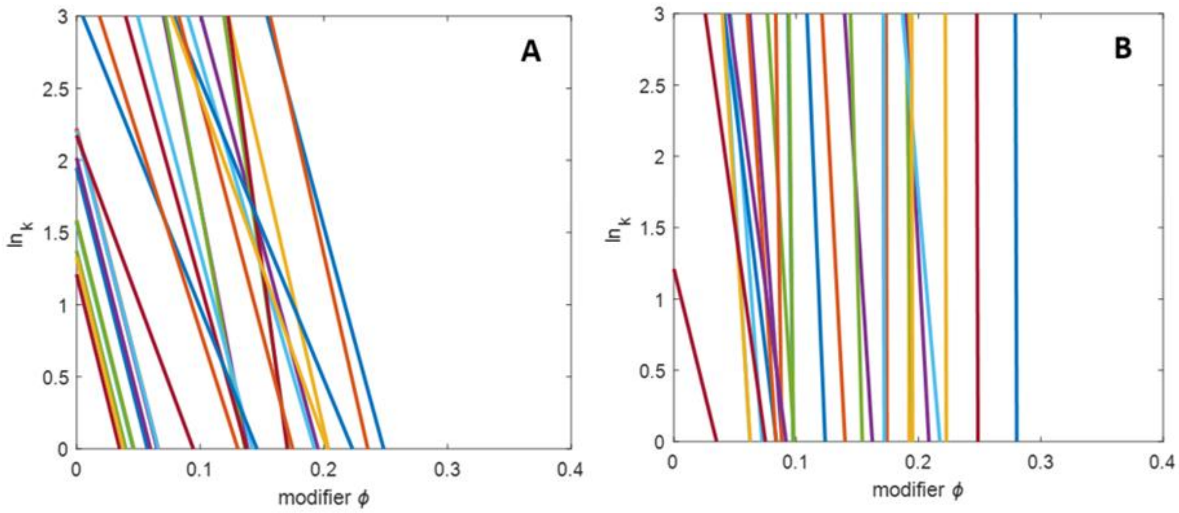


**Figure S4:** Retention-modelling plots constructed using the MOREPEAKS software by applying the LSS model to scanning-gradient data of targeted peptides for the cyano column (A) and the C18 column (B).

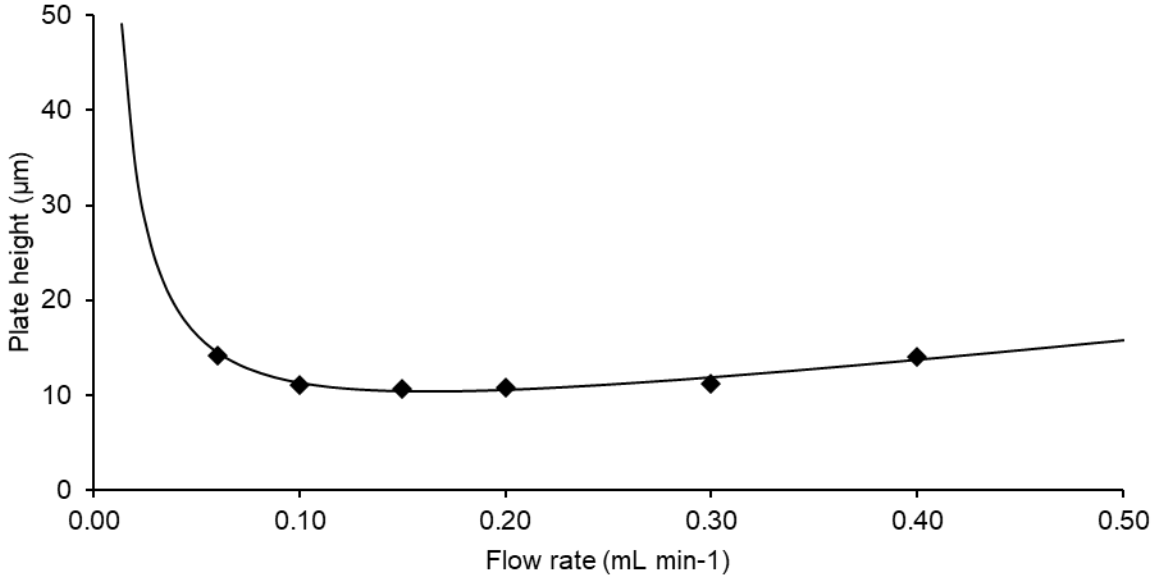


**Figure S5:** Van Deemter curve of the 150×2.1mm CN column measured using Toluene as probe molecule at 50:50 water:ACN isocratic conditions. The diamonds represent the measured datapoints and the curve is the result of fitting the A, B, and C-term of the van Deemter equation.

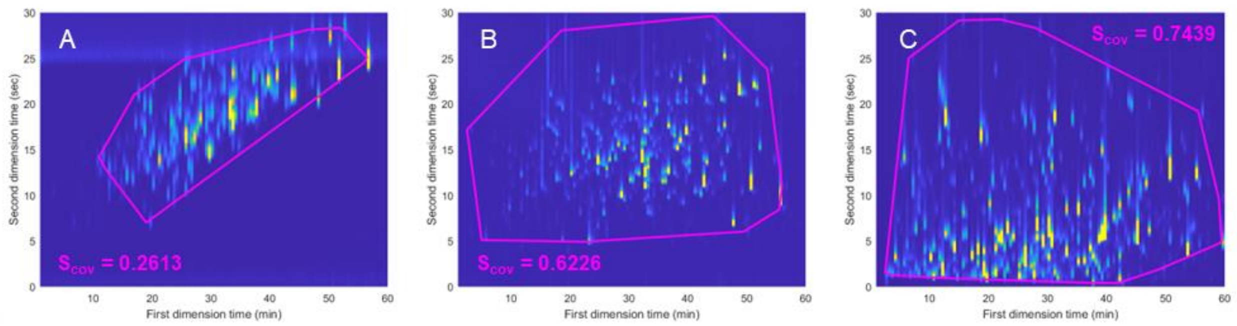


**Figure S6:** Convex Hull results (pink) plotted on top of the base-peak chromatogram (BPC) of the full (A), shifted (B) and parallel-gradient (C) LC×LC separations of the protein mixture digest sample. The resulting $S_{cov}$ values are reported in the corresponding subfigures.

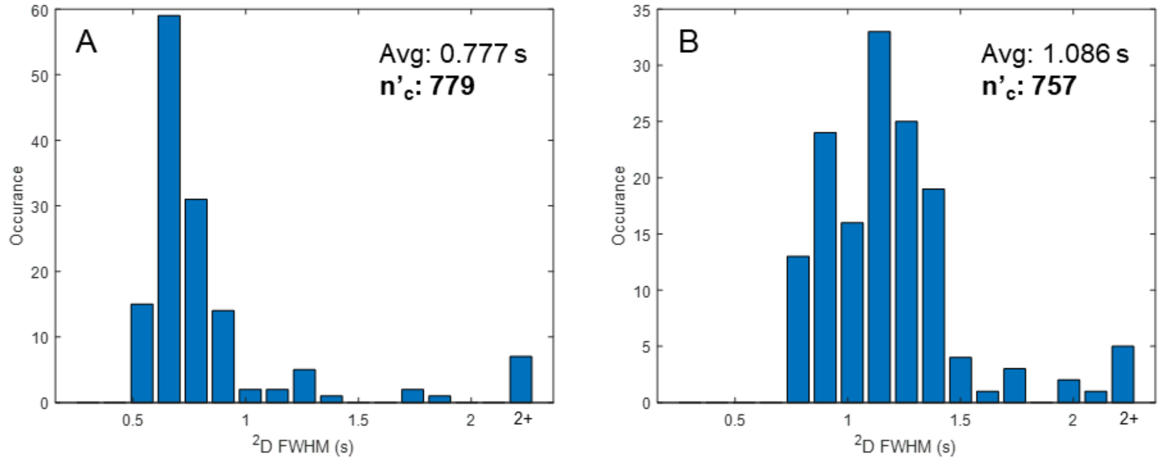


**Figure S7:** Distribution of $^{2}$D peak widths of detected features along with its average and the resulting effective peak capacity for the shifted-gradient (A) and parallel-gradient (B) methods.

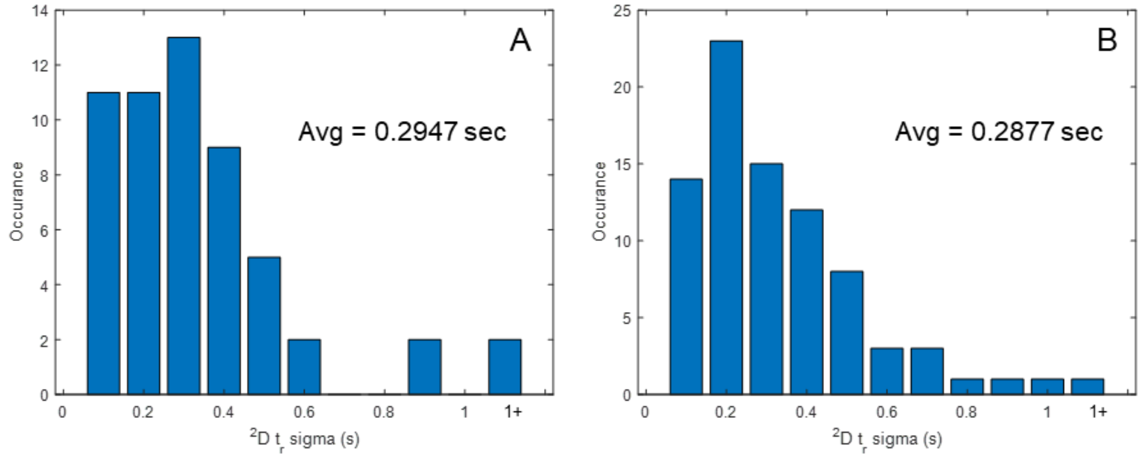


**Figure S8:** Distribution of the standard deviations (n = 4) of detected features retention times of four repeat measurements along with its average for the shifted-gradient (A) and parallel-gradient (B) methods.

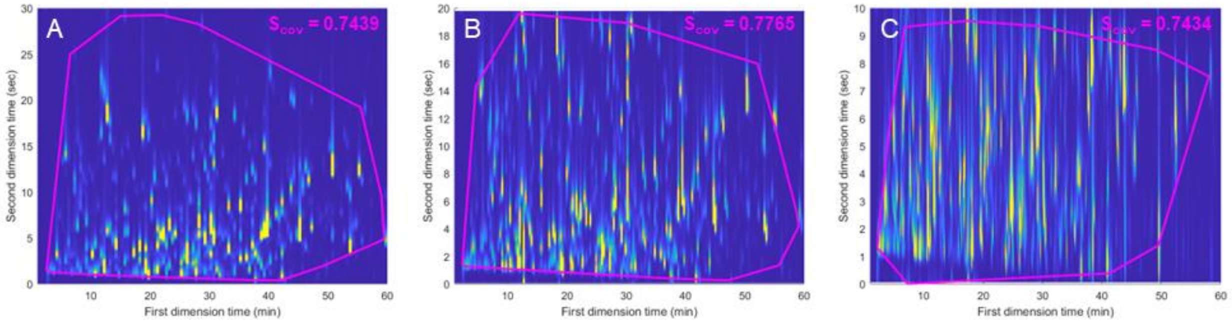


**Figure S9:** Convex Hull results (pink) plotted on top of the BPC of the parallel-gradient LC×LC separations of the protein mixture digest sample. The modulation time was changed from 30 seconds (A) to 20 (B) and 10 (A) seconds. The resulting Scov values are reported in the corresponding subfigures.

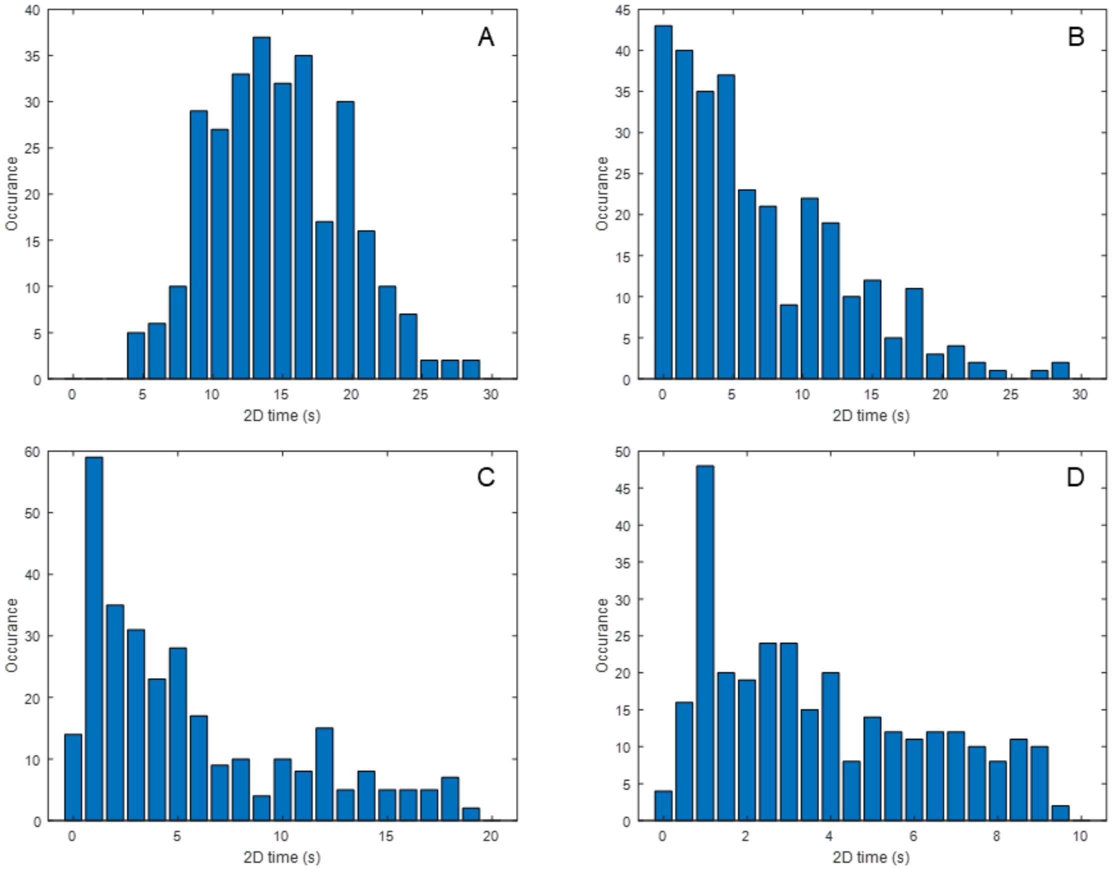


**Figure S10:** Distribution of $^{2}$D elution times of the 300 most intense peaks visible in the BPC for the shifted-gradient method (A) and for the parallel-gradient methods using 30 (B), 20 (C), and 10 (D) second modulation time.

**Table S9:** Results of duplicate cell lysate measurements using the LC-MS/MS and LC×LC-MS/MS methods.

| | C18 RPLC | | Shifted | | Parallel 30s | | Parallel 20s | | Parallel 10s | |
|---|---|---|---|---|---|---|---|---|---|---|
| | 1 | 2 | 1 | 2 | 1 | 2 | 1 | 2 | 1 | 2 |
| **MS** | 4856 | 4773 | 5872 | 5894 | 5773 | 5714 | 5160 | 5136 | 4890 | 4884 |
| **MS/MS** | 25568 | 26198 | 19312 | 19157 | 20780 | 21106 | 24425 | 24484 | 26262 | 26170 |
| **Ratio MS/MS to MS** | 5.27 | 5.49 | 3.29 | 3.25 | 3.60 | 3.69 | 4.73 | 4.77 | 5.37 | 5.36 |
| **MS/MS identified** | 6763 | 7047 | 3094 | 2917 | 4675 | 4191 | 6432 | 5971 | 8475 | 8335 |
| **MS/MS identified [%]** | 26% | 27% | 16% | 15% | 22% | 20% | 26% | 24% | 32% | 32% |
| **Average MS intensity** | 9.43E6 | 1.10E7 | 2.71E6 | 2.17E6 | 1.43E7 | 1.42E7 | 1.23E7 | 1.15E7 | 1.44E7 | 1.47E7 |
| **Peptide sequences identified** | 5858 | 6275 | 2670 | 2565 | 4142 | 3774 | 5420 | 5086 | 7177 | 7144 |
| **Protein IDs** | 1539 | 1554 | 977 | 967 | 1539 | 1554 | 1786 | 1730 | 1994 | 1989 |
| **Peaks** | 1017287 | 1002259 | 1216838 | 1192973 | 1479864 | 1472744 | 1391163 | 1386481 | 1670876 | 1734861 |
| **Peaks sequenced** | 26697 | 27402 | 20358 | 20245 | 22282 | 22589 | 25142 | 25155 | 26505 | 26463 |
| **Peaks sequenced [%]** | 2.6 | 2.7 | 1.7 | 1.7 | 1.5 | 1.5 | 1.8 | 1.8 | 1.6 | 1.5 |
| **Peaks repeatedly sequenced** | 64 | 46 | 33 | 24 | 22 | 34 | 31 | 34 | 34 | 34 |
| **Peaks repeatedly sequenced [%]** | 0.24 | 0.17 | 0.16 | 0.12 | 0.099 | 0.15 | 0.12 | 0.14 | 0.13 | 0.13 |
| **Isotope patterns sequenced [%]** | 16 | 16 | 9.7 | 9.9 | 7.9 | 7.9 | 9 | 9 | 7.6 | 7.3 |
| **Isotope patterns sequenced (z>1) [%]** | 19 | 19 | 14 | 14 | 9.9 | 9.6 | 11 | 11 | 9.9 | 9.4 |

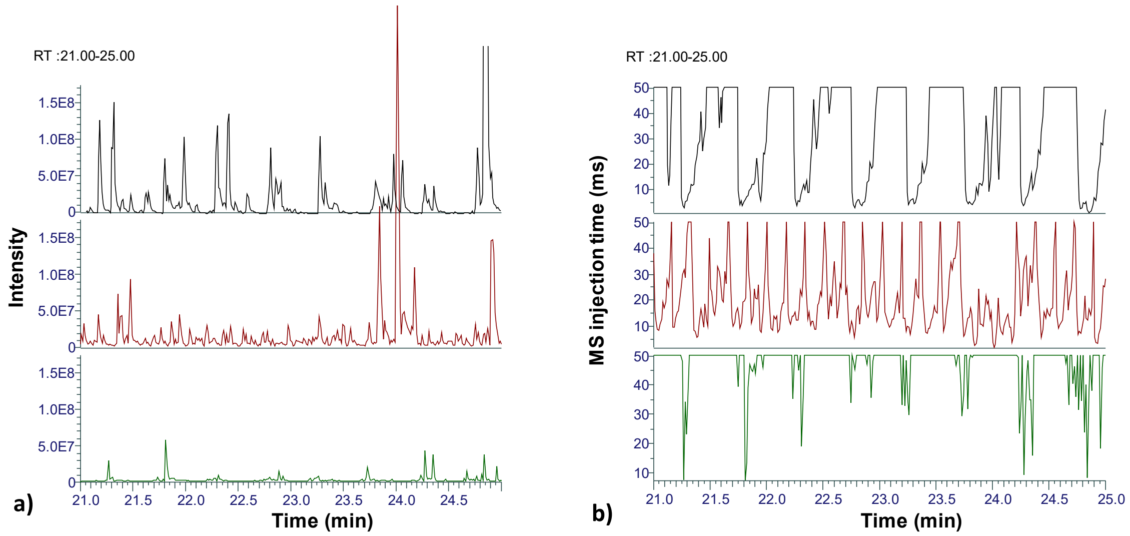


**Figure S11:** a) Detail of the MS1 base peak chromatogram from minute 21 to 25 of the parallel 30s (black), parallel 10s (red) and shifted (green) analysis of the cell lysate. b) MS1 orbitrap injection time in the same time domain as a).

## References


[1] D.R. Stoll, X. Wang, P.W. Carr, Comparison of the Practical Resolving Power of One- and Two-Dimensional High-Performance Liquid Chromatography Analysis of Metabolomic Samples, Anal. Chem. 80 (2008) 268–278. https://doi.org/10.1021/ac701676b.

[2] J.M. Davis, D.R. Stoll, P.W. Carr, Dependence of effective peak capacity in comprehensive two-dimensional separations on the distribution of peak capacity between the two dimensions, Anal. Chem. 80 (2008) 8122–8134. https://doi.org/10.1021/ac800933z.

[3] G. Semard, V. Peulon-Agasse, A. Bruchet, J.P. Bouillon, P. Cardinaël, Convex hull: A new method to determine the separation space used and to optimize operating conditions for comprehensive two-dimensional gas chromatography, J. Chromatogr. A. 1217 (2010) 5449–5454. https://doi.org/10.1016/j.chroma.2010.06.048.

[4] G. Vivó-Truyols, S. Van Der Wal, P.J. Schoenmakers, Comprehensive study on the optimization of online two-dimensional liquid chromatographic systems considering losses in theoretical peak capacity in first- and second-dimensions: A pareto-optimality approach, Anal. Chem. 82 (2010) 8525–8536. https://doi.org/10.1021/ac101420f.

[5] A.F.G. Gargano, M. Duffin, P. Navarro, P.J. Schoenmakers, Reducing Dilution and Analysis Time in Online Comprehensive Two-Dimensional Liquid Chromatography by Active Modulation, Anal. Chem. 88 (2016) 1785–1793. https://doi.org/10.1021/acs.analchem.5b04051.
